# Forecasting user engagement and competing cascades in social media diffusion: A Hawkes-Transformer approach

**DOI:** 10.1371/journal.pone.0354472

**Published:** 2026-07-24

**Authors:** Wen Zhang, Zhe Jing, Yue Guo

**Affiliations:** 1 Center for Economic Research, Shandong University, Jinan, China; 2 Faculty of Business, The Hong Kong Polytechnic University, Hong Kong, China; 3 College of Business, Southern University of Science and Technology, Shenzhen, Guangdong, China; University of Cyprus, CYPRUS

## Abstract

Social media has evolved into a socio-technical infrastructure that shapes public attention, social interaction, and information governance. Understanding how user engagement behaviors, such as retweets, comments, and likes, collectively influence information diffusion is important for forecasting digital dynamics. Using large-scale data from Sina Weibo, this study develops a hybrid Hawkes–Transformer framework that combines the interpretability of self-exciting point processes with the predictive capacity of deep learning. The model captures both interactions within a post and competition across parallel posts within the same trending topic. Empirical results show that retweets strongly amplify diffusion through self-excitation, while comments can suppress diffusion by diverting user attention. In addition, parallel cascades tend to fragment rather than reinforce information flow. By incorporating Hawkes-estimated parameters as structured inputs into a Transformer model, the proposed approach improves predictive performance while retaining interpretability. These findings provide insights into how attention is distributed and competed for in social media environments, with implications for understanding algorithmic visibility and managing information diffusion.

## Introduction

In today’s algorithmically mediated society, social media platforms have evolved into core infrastructures of information and influence, shaping how people communicate, form opinions, and construct collective narratives. Platforms such as Sina Weibo, X (formerly Twitter), and TikTok are not merely communication tools but complex socio-technical ecosystems where individuals, organizations, and algorithms continuously interact to co-create visibility, legitimacy, and meaning [1,2]. Through design affordances and algorithmic curation, these platforms govern what information is seen, shared, and amplified, thereby exerting growing influence over social discourse and market dynamics [3,4].

Within this context, a significant body of information systems (IS) and social science research has investigated how user engagement behaviors shape information diffusion and online visibility [5–8]. Yet, most studies treat these behaviors—retweets, likes, and comments—as independent and isolated activities, overlooking their interdependent and dynamic nature. In reality, engagement types interact to co-determine the trajectory of content visibility and attention allocation on social media. For instance, comments may stimulate discussion and extend content lifespan, or conversely, suppress diffusion when signaling controversy or fatigue. Likewise, likes may enhance the perceived popularity and trustworthiness of a post, indirectly encouraging further sharing. Despite these practical and theoretical implications, the collective dynamics among engagement behaviors remain underexplored, limiting our understanding of how socio-technical interactions shape information diffusion in the digital age.

Moreover, most diffusion models assume that content streams operate independently, overlooking the reality that multiple posts on the same topic often compete for limited user attention. On platforms such as Sina Weibo, algorithmic feeds, hashtag pages, and trending lists aggregate related posts under common topics, giving rise to parallel information cascades that continuously interact—competing for visibility, fragmenting engagement, or occasionally reinforcing one another. Such attention competition is not merely a technical phenomenon but a defining feature of algorithmic public spheres, where visibility and influence are shaped by the interplay between human behaviors and automated curation. Understanding these multi-cascade dynamics is therefore critical for both platform governance and firm strategy, particularly in high-stakes contexts such as crisis communication, product launches, and misinformation control [9,10].

To examine these dynamics, we draw on a large-scale dataset from Sina Weibo, comprising 29,316 posts nested within 3,662 trending topics collected over two weeks in 2024. This empirical context enables us to analyze both intra-post engagement dynamics—the interplay among retweets, comments, and likes—and inter-post competition among parallel cascades within the same topic.

**Fig 1:**
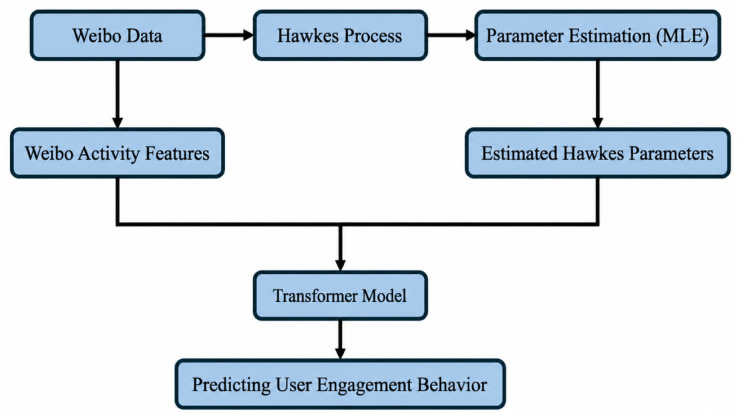
Overview of the Hawkes–Transformer framework. The model first estimates diffusion parameters using a multivariate Hawkes process, which are then combined with time-varying engagement features and input into a Transformer to predict future engagement.

Building on these data, we estimate a retweet-centric Hawkes process to capture the temporal self-excitation and cross-behavioral effects of user interactions. The estimated parameters are then integrated as structured priors into a Transformer-based prediction model, allowing the model to capture temporal dependencies and behavioral interrelations while enhancing interpretability. As illustrated in Fig 1, this hybrid Hawkes–Transformer framework links the interpretability of point-process modeling with the predictive strength of deep learning. The integration enables the model to recognize temporal dependencies and behavioral interrelations with greater transparency and accuracy. Beyond methodological innovation, the framework offers a foresight-oriented lens for anticipating how algorithmic mediation may reconfigure the future dynamics of collective attention, information visibility, and digital influence.

Our proposed model combines a retweet-centric Hawkes process—to capture self-excitation and engagement-based diffusion dynamics—with a Transformer-based deep learning model, where Hawkes-estimated parameters are incorporated as prior knowledge to enhance forecasting accuracy and interpretability. This hybrid approach bridges theory-driven modeling with data-driven prediction, allowing us to explore not only when content spreads, but how different types of engagement interact to shape diffusion outcomes.

Our analysis reveals several key findings: while retweets exhibit strong self-excitation, comments tend to suppress further diffusion—especially when they are numerous or clustered—and parallel cascades generally fragment attention rather than reinforce it. By capturing these nuanced behavioral interactions, the Hawkes–Transformer framework provides a more realistic and actionable understanding of how user behavior and algorithmic visibility co-evolve, offering implications for the future design of attention management and digital communication systems.

This study offers three main contributions to the broader literature on digital transformation and socio-technical change. First, it introduces an integrated modeling framework that explains how diverse user engagement behaviors interact and co-evolve to shape information diffusion moving beyond prior work that treats these behaviors in isolation. Second, it models competitive interactions among concurrent posts within the same topic, revealing how algorithmic visibility and attention scarcity jointly structure the dynamics of collective attention on social platforms. Third, by embedding Hawkes-estimated diffusion parameters into a Transformer-based deep learning model, the study demonstrates how theory-grounded parameters can enhance predictive performance while maintaining interpretability, offering a pathway for integrating statistical rigor with machine learning foresight in digital research.

Together, these contributions deepen our understanding of how user engagement patterns, algorithmic mechanisms, and competitive attention dynamics collectively shape information visibility in digital environments. Beyond academic insights, the findings provide governance and design foresight for firms and platforms—highlighting the importance of early-stage engagement, comment moderation, and timing strategies to sustain healthy and transparent diffusion in algorithmically mediated societies.

## Literature review

Our work draws upon and contributes to three key streams of literature: (1) the socio-economic and managerial value of social media information diffusion, which examines how digital interactions shape organizational visibility and public influence, (2) the understanding and forecasting of user engagement dynamics on social media, focusing on how behavioral and algorithmic factors jointly determine participation and diffusion, and (3) the analytical modeling of event-driven diffusion processes, particularly through point-process frameworks that capture the temporal and interactive nature of digital communication.

### The socio-economic and managerial value of social media information diffusion

Social media platforms have become transformative infrastructures that not only mediate business interactions but also shape how societies produce, circulate, and evaluate information. They influence how opinions form, brands engage with consumers, and markets respond to collective sentiment. Prior research in information systems and digital media has examined these effects from multiple perspectives, including knowledge dissemination [11–13], content virality and emotional contagion [3,14,15], platform affordances and algorithmic visibility [16], and network and misinformation dynamics [10,17]. Collectively, this stream of work demonstrates that social media is a double-edged phenomenon amplifying visibility and engagement, while creating reputational and informational risks [5,18].

However, much of the existing literature conceptualizes user engagement activities, such as retweets, likes, and comments—as independent or additive behaviors. This reductionist view overlooks how these actions dynamically interact to shape diffusion trajectories and collective attention. For instance, prior research highlights the role of emotional content in driving virality [3], yet this framework does not address how cross-behavioral interactions (e.g., commenting versus liking) alter diffusion outcomes. Similarly, existing studies illustrate how digital affordances influence visibility [16], but do not consider the competitive interplay among multiple, parallel streams of information that coexist within algorithmic feeds.

Building on this backdrop, our study advances the literature by theorizing and empirically modeling how distinct engagement activities—retweets, comments, and likes—jointly shape information diffusion and visibility within highly competitive, algorithmically curated environments. Using data from Sina Weibo’s Hot Search List, we capture the interaction and competition among parallel posts under the same trending topic, offering insights into how attention is fragmented or reinforced across concurrent cascades. This perspective extends the understanding of social media diffusion from a business performance issue to a broader socio-technical phenomenon with implications for digital governance, public discourse management, and the future sustainability of online attention economies.

### Understanding and forecasting user engagement dynamics on social media

Understanding and forecasting user engagement on social media has become a central concern in both information systems and marketing research, as these behaviors drive not only content popularity but also the broader circulation of ideas, values, and influence in digital ecosystems [19–21]. Prior studies have applied a wide range of predictive approaches, from traditional regression models [22,23] to machine learning algorithms [24–26] and deep learning architectures [27]. While these models have advanced our ability to predict engagement outcomes and assess content virality, most focus on a single type of user behavior, such as retweets or comments in isolation.

Yet, user engagement on social platforms is inherently interactive and interdependent. Different behaviors—retweeting, commenting, and liking—do not occur independently but continuously influence each other in shaping diffusion trajectories. For instance, prior research has applied deep learning to predict user interactions [28], yet it does not examine how multiple engagement types co-evolve over time. Similarly, existing studies focus on predicting virality through retweets alone [27], overlooking how comments or likes may moderate diffusion intensity or redirect user attention.

To address these limitations, our study explicitly models the dynamic interdependencies among engagement behaviors within social media posts. By integrating these interactions into a hybrid Hawkes–Transformer framework, we combine the interpretability of temporal point processes with the adaptive learning ability of deep neural networks. This integration not only improves forecasting accuracy but also yields deeper insight into the behavioral and algorithmic mechanisms driving social engagement and diffusion. Beyond predictive performance, this approach contributes to digital foresight—advancing our understanding of how engagement patterns evolve and how platforms might govern or sustain meaningful interaction in the future attention economy.

### Analytical modeling of event-driven diffusion processes

Analytical models based on temporal point processes particularly the Hawkes process, have become powerful tools for examining how events unfold and influence one another over time. In social media contexts, these models capture the temporal dependencies among engagement events such as retweets, comments, and likes, illustrating how prior interactions increase the likelihood of subsequent ones [8,29]. The Hawkes framework thus offers a theoretically grounded approach to modeling self-exciting behaviors, in which each user action generates feedback that shapes the momentum of diffusion. Researchers in information systems have applied Hawkes processes to model retweet cascades and information spread, emphasizing their ability to represent cascading and path-dependent dynamics [8,9].

However, most existing studies rely on univariate formulations that capture a single behavior—typically retweets—while overlooking how different engagement types interact simultaneously. Furthermore, information cascades are often treated as independent processes, ignoring the possibility that multiple posts on the same topic may compete or reinforce one another within algorithmic feeds. These simplifications limit our ability to understand the complex attention ecology that governs social diffusion.

Recent advances have explored hybrid approaches that integrate temporal point processes with deep learning architectures to model complex event dynamics. For example, the Recurrent Marked Temporal Point Process (RMTPP) employs recurrent neural networks to model event sequences in continuous time [30], while the Neural Hawkes Process uses continuous-time LSTM structures to learn the intensity function end-to-end [31]. More recently, Transformer-based variants such as the Transformer Hawkes Process leverage self-attention mechanisms to capture long-range temporal dependencies in event sequences [32]. More recent work continues to extend this line of research by exploring more flexible neural architectures for modeling temporal dependencies in event sequences [33]. However, these approaches generally focus on end-to-end learning of the intensity function.

Extending this line of research, we develop a retweet-centric multivariate Hawkes framework that incorporates multiple engagement behaviors—retweets, comments, and likes—together with cross-cascade competition among parallel posts. In addition, we adopt a two-stage design in which a multivariate Hawkes model is first estimated to capture interpretable behavioral dynamics, and the resulting parameters are incorporated as structured inputs into a Transformer-based prediction model. Rather than learning the intensity function end-to-end, this design emphasizes interpretability and the explicit modeling of cross-cascade competition, providing a more comprehensive depiction of digital diffusion as an interdependent, event-driven process.

### Summary and research gap

Prior research on social media information diffusion has provided valuable insights into content virality, engagement forecasting, and event-based diffusion modeling using frameworks such as the Hawkes process. Yet, two critical gaps remain largely unexplored. First, most studies treat engagement behaviors—such as retweets, comments, and likes—as isolated mechanisms, overlooking their interdependent and evolving nature in shaping diffusion trajectories. Second, existing models often assume that information cascades operate independently, neglecting the competitive and reinforcing interactions that occur among parallel posts under the same topic. These limitations constrain our understanding of how user behaviors collectively generate, sustain, or fragment public attention within algorithmically curated environments.

To address these gaps, this study proposes a retweet-centric modeling framework that integrates multiple engagement behaviors and cross-cascade interactions between parallel information cascades. By embedding Hawkes-estimated parameters into a Transformer-based deep learning architecture, the model enhances both forecasting capability and theoretical interpretability, bridging data-driven prediction with behaviorally grounded diffusion theory. Using large-scale data from Sina Weibo, we empirically validate this approach and uncover how cross-behavioral interdependencies and cascade competition shape collective attention.

Beyond extending prior IS and diffusion research, our findings contribute to technological forecasting and social change by illuminating the mechanisms through which algorithmic mediation structures visibility, amplifies or constrains engagement, and influences the sustainability of attention in digital societies. These insights offer foresight for digital governance and strategic content management, helping platforms and organizations design healthier and more transparent attention ecosystems.

## The social media platform: Sina Weibo

Launched in 2009, Sina Weibo is one of China’s largest social media platforms, often compared to X due to its microblogging format and real-time content ecosystem. As of late 2024, Sina Weibo reported over 580 million monthly active users, solidifying its role as a central hub for public discussions, news dissemination, and digital marketing activities. Users create posts and engage with them through retweets, comments, and likes, fostering real-time interactions and driving content virality.

**Fig 2:**
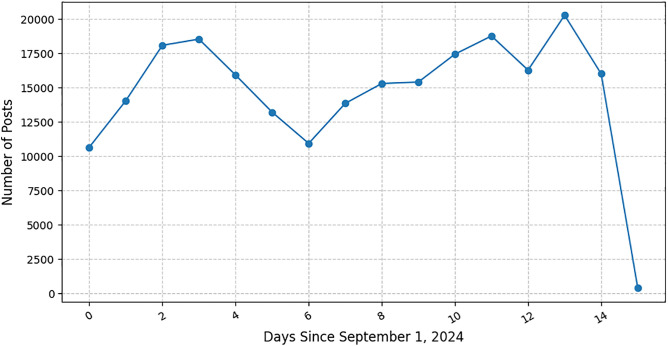
Daily number of posts in Sina Weibo trending topics (September 1–15, 2024). The figure shows fluctuations in the volume of posts associated with trending topics over time.

In addition to these engagement mechanisms, Weibo features the Hot Search List (see Table 1 for an example), a real-time hot topic ranking system that identifies the top 50 trending topics based on user searches and discussions. As an official tool of the platform, the Hot Search List reflects issues of widespread societal interest by capturing intensive public attention and sustaining user discussions. Its ranking algorithm evaluates metrics such as post volume, reading volume, and engagement levels to generate a comprehensive heat score, with the final list updating every minute to reflect discussion activity and content dissemination intensity. Topics that attract widespread public attention and sustained discussion are more likely to stay on the list. As shown in Fig 2, the number of trending topics ranges from 10,500–20,300 per day, with an average of approximately 15,700 topics, but only 50 topics are displayed on the Hot Search List at any given time, highlighting the intense competition for user attention on social media.

**Table 1:** Sina Weibo hot search list.

1. Duke the Cat is so Strong (1,167,571 discussions, Hot) 2. Why Do Ninth Graders Return Home Early? (524,598 discussions, Hot) 3. China’s Spring Festival Traditions Are Reaching the World (497,738 discussions) 4. Blackpink’s Lisa’s Parents and Brother Have Passed Away (494,370 discussions) 5. Is a Snake the Main Character of a Medical School Emblem? (472,822 discussions) 6. …….

To better capture the dynamics of user engagement within this platform environment, we introduce an additional construct “active user”, defined as the number of unique users engaging with a post within a given time interval. Retweet volume measures the intensity of engagement but does not reflect its breadth across distinct users. Active users capture the extent of audience reach and exposure diversity, reflecting how widely content spreads across individuals. This distinction is consistent with prior research on information diffusion, which emphasizes both the depth and breadth of cascades [10]. From an epidemiological perspective, active users approximate the size of the “susceptible population” at a given time, providing a complementary measure of cascade breadth relative to retweet-based depth. As such, active users provide an alternative view of information diffusion by capturing the dispersion of engagement across users. This distinction is particularly important in our empirical setting, where diffusion dynamics are shaped not only by repeated engagement but also by how widely attention is distributed across users.

**Fig 3:**
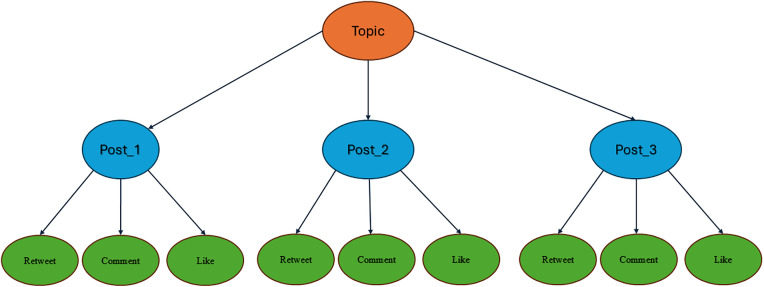
Hierarchical structure of user engagement on Sina Weibo. The structure consists of trending topics, posts within each topic, and user engagement behaviors.

**Fig 4:**
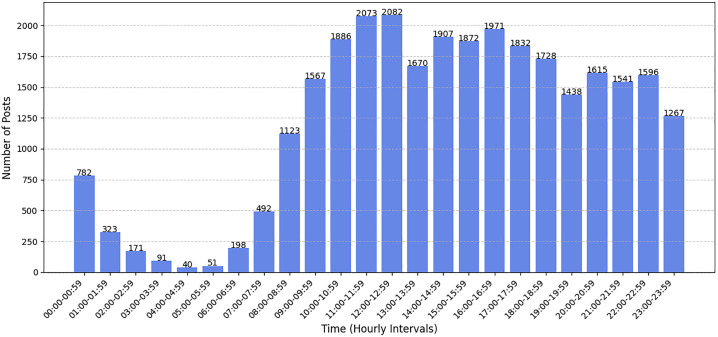
Distribution of post activity over a 24-hour period. The figure shows the number of posts published at each hour, illustrating temporal patterns in user activity.

To further illustrate how user engagement unfolds within this environment, Fig 3 depicts the hierarchical structure of interactions on Sina Weibo. At the top level, each trending topic links to a dedicated page displaying posts tagged with the corresponding hashtag, serving as a focal point for aggregated discussions. For each post, users can retweet, comment, and like, collectively shaping the visibility and diffusion trajectory of the information. Fig 4 shows that post activity peaks between 10:00 AM and 10:00 PM, with notable spikes around noon and early evening, aligning with typical break and leisure periods.

In sum, these features collectively make Sina Weibo’s Hot Search List an ideal context for exploring how multiple posts under the same trending topic interact and compete for user attention. This environment allows us to examine how engagement activities—such as retweets, comments, and likes—not only drive post visibility but also shape the trajectory of information diffusion, offering insights into the evolution of competing and complementary information streams in dynamic social media ecosystems [13,34].

## Data collection

To construct the study sample, we collected all topics that appeared at least once on the Sina Weibo Hot Search List between September 1, 2024, and September 15, 2024. The selection of this two-week period reflects an empirical trade-off between ensuring a sufficiently large sample for robust analysis and maintaining computational feasibility. A shorter observation window would cover limited trending topics, limiting the generalizability of our findings. Conversely, a longer window would significantly increase computational complexity, posing challenges for model estimation. We collected the data from publicly accessible content on Sina Weibo using API-based procedures. Our final dataset includes 3,662 trending topics from the Sina Weibo Hot Search List, along with 29,316 associated posts that appeared under these topics during the observation period. To further characterize the structure of parallel cascades, we report the distribution of posts per topic in Table 2.

**Table 2:** Descriptive statistics of cascade structure.


Posts per topic	Number of topics	Percentage
1 (no cross-cascade)	215	5.9%
2–5	1,303	35.6%
6–10	1,167	31.9%
11–20	847	23.1%
>20	130	3.5%
Total	3,662	

For each post, we extracted all associated engagement metrics: retweets (measuring the breadth of post dissemination across user networks), comments (capturing the depth of user discussion and interaction), and likes (indicating passive engagement and audience interest). In addition, we recorded timestamps for posts, retweets, and comments to track the temporal dynamics of information diffusion. Notably, Sina Weibo does not provide timestamps for likes, which prevents us from directly modeling their temporal patterns. To bypass this limitation and capture the influence of likes on the future retweets, we incorporate likes into the baseline intensity of our model rather than modeling them as discrete events, as discussed in the following section. This is reasonable since the number of likes primarily reflects cumulative audience interest rather than active propagation behavior, which makes them more suitable for incorporation into the baseline intensity rather than as individual, time-dependent events.

**Fig 5:**
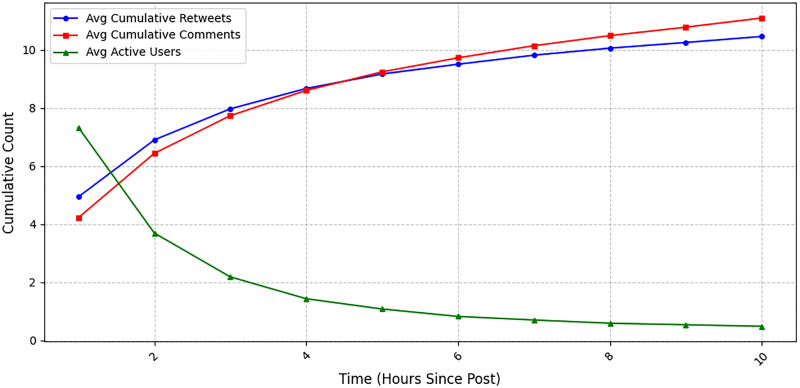
Temporal evolution of cumulative engagement. The figure shows the average cumulative retweets, comments, and active users over the 10-hour observation window.

We restricted the observation window to ten hours following each post’s publication. This choice is supported by model-free evidence, as shown in Fig 5, which highlights the temporal concentration of user interactions within the initial hours after a post’s emergence.

Specifically, cumulative retweets and comments rise sharply within the first ten hours, indicating that the vast majority of user interactions occur during this early window. Beyond this point, engagement growth stabilizes, with diminishing marginal increases in retweets and comments, suggesting that content diffusion reaches saturation relatively quickly. Additionally, user participation declines sharply after the first four hours, further reinforcing the critical role of early-stage interactions in shaping the overall information diffusion process. Thus, we believe that this ten-hour window effectively balances capturing the essential dynamics of information diffusion and ensuring computational feasibility, as extending the observation period would provide limited additional insights while significantly increasing computational complexity.

## Multivariate engagement event model for information diffusion

In many stochastic processes, events occur at discrete points in time or space, and point process models provide a stochastic framework for capturing the random distribution and temporal dependencies among these events. This study focuses on information diffusion on social media platforms, particularly Sina Weibo, where users engage with content by posting, retweeting, commenting, and liking that key mechanisms that drive information diffusion.

To capture the diffusion dynamics of trending topics, we develop a retweet-centric self-exciting process with cross-cascade effects and embedded comment and like effects, which explicitly models how different types of engagement activities influence the likelihood of retweets. Specifically, the model captures: (1) the self-exciting effect of retweets, where past retweets increase the likelihood of future retweets; (2) the indirect effects of comments and likes on retweet behavior; and (3) cross-cascade effects, where retweets within parallel posts influence the retweet likelihood of the focal post under the same trending topic. This model enables the quantification of how self-excitation, cross-engagement, and cross-cascade effects collectively shape the diffusion process across parallel information streams on social media.

### Point process and conditional intensity function

A point process can be represented by a counting process $N^{i}(t)$, which denotes the cumulative number of retweet events for post i up to time t. This counting process is right-continuous, non-decreasing, and takes non-negative integer values, with each engagement event incrementing $N^{i}(t)$ by one. If the first engagement occurs at time $t_{\text{1}}\;$and the nth engagement occurs $t_{n}$, then $N^{i}\left(t_{n}\right)-N^{i}\left(t_{\text{1}}\right)\;$represents the number of engagement events within the interval $\left(t_{\text{1}},t_{n}\right)$. In our empirical setting, we construct incremental engagement counts by measuring the number of new retweets within each one-hour interval, denoted as $\Delta N^{i}(t).$

In the context of Sina Weibo, user engagement events are not independent; each retweet event influence the content’s visibility and perceived popularity, thereby increasing or decreasing the likelihood of future engagements. This temporal dependency among engagement events can be effectively captured using a self-exciting point process, which explicitly models how past events influence the arrival of future events. A key distinguishing feature of point processes is their conditional intensity function λ(t), which represents the instantaneous rate at which new events are expected to occur at time t, given the history of past events. In a self-exciting point process, the conditional intensity depends not only on time but also on the cumulative history of prior events, allowing the model to capture self-reinforcing dynamics in user engagement activities. Specifically, the conditional intensity $\lambda^{i}(t)$ can be interpreted as the instantaneous diffusion rate of content i, representing the probability that a new event occurs at time t, given the history of past retweet events, while comment activity enters as an influencing factor on the retweet intensity.

Unlike traditional multivariate Hawkes processes, which typically construct separate intensity functions for each engagement type (e.g., $\lambda^{retweet}(t)\;$and $\lambda^{comment}(t)$), this study adopts a retweet-centric approach. Specifically, we define a single conditional intensity function $\lambda^{i}(t)$ for information diffusion, focusing on the occurrence of retweet events as the primary indicator of content diffusion. In this framework, comments do not have an independent intensity function. Instead, their influence is incorporated into the retweet intensity function, capturing their potential role in affecting future retweet likelihood. This modeling approach simplifies the representation of engagement dynamics while preserving the essential interactions between retweets and comments, aligning with the core nature of social media information diffusion, which is directly driven through retweets.

Given that Sina Weibo does not provide timestamps for likes, we are unable to directly model the temporal dynamics of likes using a point process framework. Instead, we incorporate the cumulative number of likes into the baseline intensity μᵢ, treating it as a post-specific constant rather than a time-varying component. This formulation allows μᵢ to differ across posts based on observed audience engagement, as shown below:


$$\begin{array}{c} \mu^{i}=\mu_{\text{0}}+\gamma {Likes}^{i} \end{array}$$

where ${Likes}^{i}\;$represents the total number of likes for post *i* observed at the end of the 10-hour observation window and is treated as a time-invariant scalar. Before incorporating likes, the baseline intensity reduces to $\mu^{i}\;$= μ₀. The term μ₀ captures inherent differences in post visibility, such as variation in the poster’s social network size. The second term, $\gamma {Likes}^{i}$, reflects the overall level of audience interest associated with the post.

Finally, the conditional $\lambda^{i}\left(t|H_{t}^{i}\right)\;$is interpreted as the diffusion rate of information and is defined as follows:


$$\begin{array}{c} \lambda^{i}\left(t|H_{t}^{i}\right)=\underset{\Delta t\to \text{0}}{\text{lim}}\frac{E\left\{N^{i}(t,t+\Delta t]|H_{t}^{i}\right\}}{\Delta t} \end{array}$$

$$\begin{array}{c} \begin{array}{ccc} & & =\underset{\Delta t\to \text{0}}{\text{lim}}\frac{\left\{N^{i}(t+\Delta t)-N^{i}(t)>\text{0}|H_{t}^{i}\right\}}{\Delta t} \end{array} \end{array}$$ (1)

where $\lambda^{i}\left(t|H_{t}^{i}\right)$ is a non-negative conditional intensity function, and $H_{t}^{i}$ represents the history of past retweet events for post i up to time t, while comment activity is incorporated as an influencing factor on the retweet intensity.

### Multivariate self-exciting process

The self-exciting process is a fundamental model in point process theory, where past events increase the likelihood of future events by raising the conditional intensity function. This formulation assumes that the impact of historical events decays monotonically over time, typically following an exponential decay function, but accumulates additively to reinforce future event occurrences.

In the context of social media information diffusion, retweets serve as the primary mechanism driving content spread. Each retweet expands a post’s audience by introducing it to new users, thereby increasing visibility and accelerating further engagement. Since information propagation depends on reaching larger networks, retweets directly contribute to the self-reinforcing diffusion process, a classic self-exciting phenomenon.

While retweets consistently promote diffusion, comments can have a dual effect. High comment volume may enhance content visibility and increase the chances of further retweets. However, critical or controversial comments may signal content saturation or generate negative sentiment, reducing retweet likelihood. Additionally, comments may shift user focus from spreading content to discussing it, potentially slowing down the diffusion process.

Traditional multivariate Hawkes models typically assign independent intensity functions to different engagement types (e.g., $\lambda^{retweet}(t)\;$and $\lambda^{comment}(t)$), modeling them as parallel but separate processes. However, in reality, retweets and comments are inherently interdependent, jointly shaping content visibility and information spread.

To better capture this interdependence of engagement activities influencing retweet behavior, our model adopts a retweet-centric self-exciting and cross-cascade mutual-exciting process. In this model, retweets serve as the primary self-exciting mechanism, directly driving information diffusion by expanding a post’s audience reach. Meanwhile, comments are incorporated as an additional engagement mechanism, potentially influencing the visibility of a post and its likelihood of being retweeted. Rather than modeling comments as an independent process, their impact is embedded into the retweet intensity function to better capture their influence on retweet behavior.

Specifically, the self-exciting point process models information diffusion by capturing how retweet events, together with comment activity, influence the likelihood of future retweets. Therefore, in addition to the dynamic baseline intensity $\mu^{i}(t)$, we introduce two self-excitation components, as shown in Equation (2).

**Fig 6:**
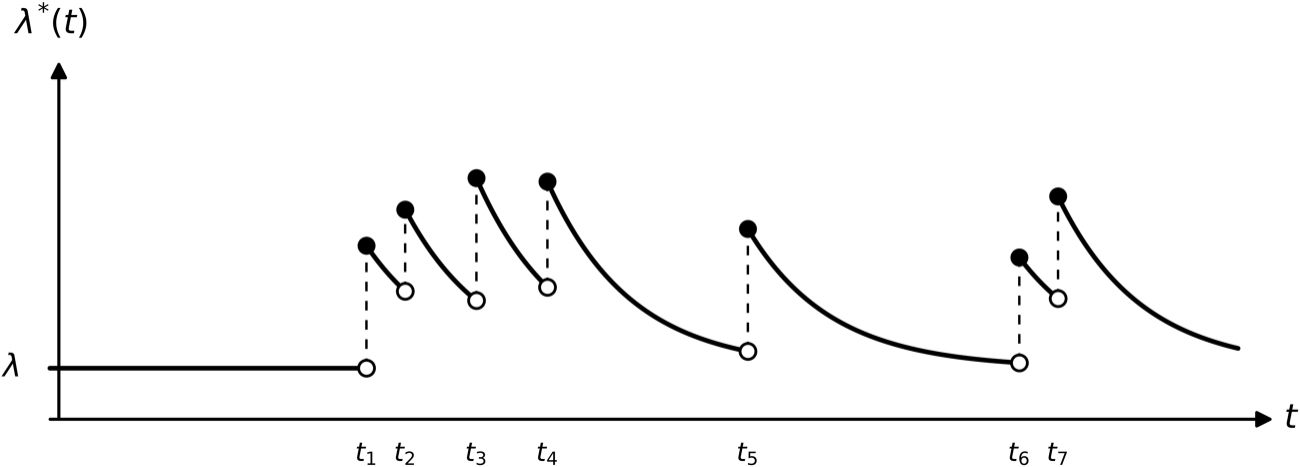
Illustration of the self-exciting point process. Each past retweet event generates an excitatory effect that decays exponentially over time, contributing to the cumulative conditional intensity.

First, retweet events trigger self-excitation, reinforcing information diffusion over time. Each retweet event at time $t_{k\;}^{i}$ contributes an additional positive excitatory effect that decays exponentially as time progresses. This effect is not permanent but gradually decays over time (as shown in Fig 6). This decay is modeled using an exponential kernel function:$g^{i}(T-t)=\alpha^{i}e^{-\beta^{i}(T-t)}$, with $\alpha^{i}>\text{0}$ and $\beta^{i}$ > 0.

Second, unlike traditional multivariate Hawkes models that assign an independent intensity function to comments, our model embeds their effect within the retweet intensity function. In this way, comments are incorporated as an additional self-excitation component that may indirectly influence content diffusion by affecting the likelihood of retweets. Specifically, a comment event at time $t_{m\;}^{i}$ also generates an effect for future retweets, which may be either excitatory or suppressive, and follows a similar exponential decay function. The resulting conditional intensity function is expressed as:


$$\begin{array}{c} \lambda^{i}\left(t|H_{t}^{i}\right)=\mu^{i}+\sum_{t_{k}^{i}<t}\alpha_{\text{1}}^{i}e^{-\beta_{\text{1}}^{i}\left(t-t_{k}^{i}\right)}+\sum_{t_{m}^{i}<t}\alpha_{\text{2}}^{i}e^{-\beta_{\text{2}}^{i}\left(t-t_{m}^{i}\right)} \end{array}$$ (2)

where $\mu^{i}$>0, and all past retweet and comment times satisfy $t_{k}^{i},t_{m}^{i}<t$. The parameters $\beta_{\text{1}}^{i},\beta_{\text{2}}^{i}>\text{0}$. The retweet-induced parameter $\alpha_{\text{1}}^{i}\geq \text{0}$captures the self-excitation effect of retweets, such that each past retweet increases the likelihood of future retweets. In contrast, the comment-induced parameter $\alpha_{\text{2}}^{i}$is not sign-restricted, allowing for both reinforcing ($\alpha_{\text{2}}^{i}>\text{0}$) and suppressive ($\alpha_{\text{2}}^{i}<\text{0}$) effects, depending on whether comments promote further diffusion or divert user attention away from retweeting.

To ensure the stability of the model, we require the constraint $\alpha_{\text{1}}^{i}/\beta_{\text{1}}^{i}<\text{1}$, preventing the self-excitation process from diverging. This ensures that information diffusion gradually stabilizes rather than growing indefinitely. Specifically, $\alpha_{\text{1}}^{i}$measures the magnitude of self-excitation from retweets, while $\alpha_{\text{2}\;}^{i}$captures the directional influence of comments on retweet behavior. The parameters $\beta_{\text{1}}^{i}$and $\beta_{\text{2}}^{i}\;$govern how quickly the impact of past retweet and comment events fade over time. As a result, the diffusion rate of information for post i at time t depends on the cumulative effect of all past retweets, comments, and likes, allowing us to model engagement-driven content propagation within an integrated modeling framework.

### Multivariate self-exciting process with cross-cascade effects

While the self-exciting process effectively models how engagement events within a single post influence retweet behavior, real-world social media dynamics are often more complex. In particular, parallel information streams comprising multiple posts under the same trending topic occur simultaneously. These parallel posts compete for user attention, and retweets of one post can influence the retweet dynamics of others. To better capture this complexity, we extend the model to analyze cascade diffusion mechanisms on Sina Weibo.

In the specification of the model, parallel cascades are operationally defined as posts belonging to the same trending topic that are active within the same 10-hour observation window. For each focal post, parameters are estimated independently using its own event history. Cross-cascade effects are incorporated by conditioning on the retweet histories of other posts within the same topic, which enter the intensity function as exogenous inputs rather than through pairwise parameterization.

In addition to the self-excitation components, as shown in Equation (3), we incorporate Jparallel information flows (i.e., posts under the same trending topic) that compete for user attention. For each parallel post j≠i, let $\left\{t_{j}^{l}\right\}$denote the sequence of retweet event times associated with post j, where lindexes individual retweet events. These retweet events from post jmay influence the diffusion intensity of the focal post i. In this formulation, parameters are estimated at the post level, with each post having its own parameter vector.

Cross-cascade effects are incorporated by conditioning on the retweet histories of other posts within the same topic, which enter the intensity function as exogenous inputs rather than through pairwise parameterization. The cross-cascade component is defined as a double summation. The outer summation runs over all parallel posts j≠i within the same trending topic, while the inner summation aggregates all historical retweet events $t_{j}^{l}<t$ from post j. This structure captures the cumulative influence of retweet activity from competing posts on the focal post’s diffusion intensity.

The complete model is expressed as:


$$\begin{array}{c} \lambda^{i}(t|H_{t}^{i})=\mu^{i}+\sum_{t_{k}^{i}<t}\alpha_{\text{1}}^{i}e^{-\beta_{\text{1}}^{i}(t-t_{k}^{i})}+\sum_{t_{m}^{i}<t}\alpha_{\text{2}}^{i}e^{-\beta_{\text{2}}^{i}(t-t_{m}^{i})}+\sum_{j\neq i}\sum_{t_{j}^{l}<t}\alpha_{\text{3}j}^{i}e^{-\beta_{\text{3}j}^{i}(t-t_{j}^{l})} \end{array}$$ (3)

This formulation implies that the diffusion intensity of post *i* depends not only on its own engagement history (retweets and comments), but also on the cumulative retweet activity of competing posts within the same topic. The parameter $\alpha_{\text{3}j}^{i}$ represents the impact of the j-th parallel post cascade on the diffusion intensity of the focal post i. Unlike the self-excitation parameter $\alpha_{\text{1}}^{i}$, the cross-cascade parameter $\alpha_{\text{3}j}^{i}$ is not sign-restricted, allowing for both reinforcing and suppressive interactions across parallel posts. A positive $\alpha_{\text{3}j}^{i}$ indicates that retweet activity from post j increases the diffusion intensity of post i, reflecting cross-cascade amplification. In contrast, a negative $\alpha_{\text{3}j}^{i}$ suggests competition for user attention, whereby activity in post j reduces the retweet likelihood of the focal post i. We summarize the constraints and interpretations of all model parameters in Table 3.

**Table 3:** Summary of parameter constraints and interpretations.

Parameter	Constraint	Interpretation
μᵢ	≥ 0	Baseline intensity
γ	≥ 0	Effect of cumulative likes on baseline intensity
β	> 0	Decay rate
α₁ (retweet)	≥ 0	Self-excitation
α_2_ (comment)	Unrestricted	Reinforcing or suppressive effect
α₃ⱼ (cross)	Unrestricted	Cross-cascade interaction (competition or amplification)

In our setting, parallel cascades are defined as posts belonging to the same Hot Search topic that are active within the same 10-hour observation window. All model parameters are estimated at the post level, with each post having its own parameter vector. Cross-cascade effects are incorporated by conditioning on the retweet histories of other posts within the same topic, which enter the intensity function as exogenous inputs rather than through pairwise parameterization. This design ensures that the model captures cross-cascade interactions without introducing a combinatorial increase in the number of parameters.

### Model estimation procedure and results

In our study, we employ maximum likelihood estimation (MLE) to infer the parameters of our Hawkes model. The estimation is implemented using the L-BFGS-B algorithm with box constraints. Parameters are initialized at μ₀ = 0.1, α = 0.5, β = 1.0, and γ = 0.01. The optimization terminates when the gradient norm falls below 1e-5 or after a maximum of 1,000 iterations, whichever occurs first.

Specifically, we analyze the sequence of observed event times $\left\{t_{\text{1}},\ldots ,t_{n}(T)\right\},\;$where $t_{n}(T)\;$represents the time of the last retweet event within the interval $\left(t_{k},T\right].$ The objective is to construct and maximize the likelihood function based on these observed data points, enabling us to accurately capture the dynamics of information diffusion in social media networks. As shown in Equation (4), the joint density function L is formulated to reflect the probability of the observed sequence of retweet events, incorporating the influence of past retweet events on future occurrences. This approach provides a robust framework for understanding and predicting user engagement behavior in social media.


$$\begin{array}{c} L=f\left(t_{\text{1}},t_{\text{2}},\ldots ,t_{n(T)}\right)=\prod_{i=\text{1}}^{n(T)}f\left(t_{i}\right) \end{array}$$ (4)

From $\lambda \left(t_{i}\right)=\frac{f\left(t_{i}\right)}{\text{1}-F\left(t_{i}\right)}$, we can derive $f\left(t_{i}\right)=\lambda \left(t_{i}\right)\text{exp}\left(-\int_{t_{k}}^{t_{i}}\lambda (s)ds\right)$. Substituting this into Equation (4), the likelihood function is expressed as:


$$\begin{array}{c} L=\prod_{i=\text{1}}^{n(T)}f\left(t_{i}\right)=\prod_{i=\text{1}}^{n(T)}\lambda \left(t_{i}\right)\text{exp}\left(-\int_{t_{i-\text{1}}}^{t_{i}}\lambda (s)ds\right) \end{array}$$

$$\begin{array}{c} \begin{array}{c} =\left[\prod_{i=\text{1}}^{k}\lambda \left(t_{i}\right)\right]\text{ex}\text{p}\left(-\int_{\text{0}}^{t_{n(T)}}\lambda (s)ds\right) \end{array} \end{array}$$ (5)

Typically, the log form of the likelihood function is easier to maximize, denoted as l(θ;t)=ln(L(θ;t)) Therefore, based on the observed data of retweet events, the log-likelihood function for estimating the parameter set $\hat{\theta }\;$is:


$$\begin{array}{c} L=-\int_{\text{0}}^{t_{n(T)}}\lambda (s)ds+\sum_{i=\text{1}}^{k}\text{log}\lambda \left(t_{i}\right) \end{array}$$ (6)

During the model fitting process, we analyzed the timestamps of posts, retweets, and comments for 29,316 posts. However, for 1,097 posts, the estimation procedure did not converge within a reasonable time frame. As these cases constitute only 3.74% of the dataset, we excluded them from the final analysis to ensure estimation reliability. Given the small proportion of excluded cases, their omission is unlikely to impact the accuracy of the parameter estimates. Consequently, the final sample used for parameter estimation consists of 28,219 posts.

Table 4 presents the descriptive statistics for the estimated parameters of the Hawkes model, denoted as $\theta^{i}=(\mu_{\text{0}},\gamma ,\alpha_{\text{1}}^{i},\alpha_{\text{2}}^{i},\alpha_{\text{3}}j^{i},\beta_{\text{1}}^{i},\beta_{\text{2}}^{i},\beta_{\text{3}}j^{i}),j=\text{1},\ldots ,J$. These estimates provide insights into the dynamics of information diffusion on Sina Weibo. The average baseline intensity $\mu_{\text{0}}$is 0.362, capturing the inherent likelihood of engagement in the absence of prior triggering events. The relatively large dispersion (std. dev. = 0.831) indicates substantial heterogeneity in baseline popularity across posts. The parameter γ, with a mean of 0.035, captures the marginal effect of accumulated likes on the baseline diffusion intensity.

**Table 4:** Descriptive statistical analysis of Hawkes model.

Parameter	Mean	Median	Std. Dev.	Min.	Max.
$\mu_{\text{0}}$	0.362	0.083	0.831	0.001	26.729
γ	0.035	0.009	0.083	0.000	2.671
$\alpha_{\text{1}}^{i}$	0.655	0.418	0.563	0.001	7.267
$\beta_{\text{1}}^{i}$	3.430	1.505	3.453	0.100	10.000
$\alpha_{\text{2}}^{i}$	−0.412	−0.253	1.388	−7.664	6.785
$\beta_{\text{2}}^{i}$	0.667	0.531	0.616	0.100	10.000
$\alpha_{\text{3}j}^{i}$	−0.193	−0.060	0.861	−7.662	6.833
$\beta_{\text{3}j}^{i}$	0.859	0.590	0.880	0.100	9.845

Specifically, each additional like is associated with an increase of approximately 0.035 units in the baseline intensity. Because the distribution of likes is highly right skewed, we further illustrate this effect using empirical percentiles of the like distribution and the median estimated γ value reported in Table 4 (0.009). In our sample, the median post receives 106 likes, the 75th percentile receives 468 likes, and the 90th percentile receives 1,927 likes. Moving from the median to the 75th percentile corresponds to an increase of 362 likes and an increase of approximately 3.26 units in the post-specific baseline intensity. Moving from the median to the 90th percentile corresponds to an increase of 1,821 likes and an increase of approximately 16.39 units, holding other factors constant. This suggests that audience validation, as reflected by likes, plays a meaningful role in enhancing content visibility.

For self-excitation effects, the mean value of $\alpha_{\text{1}}^{i}\;$is 0.655, indicating that retweets strongly reinforce subsequent retweet activity. This effect varies substantially across posts, as reflected by the wide range of values. The corresponding decay rate $\beta_{\text{1}}^{i\;}\;$has a mean of 3.430, implying that the influence of past retweets decays relatively quickly over time, leading to a rapid decline in marginal impact. To further characterize the stability of the excitation process, we compute the self-excitation ratio $\alpha_{\text{1}}^{i}/\beta_{\text{1}}^{i}$ at the post level. The results show that this ratio is consistently below 1, with a mean of 0.29 (SD = 0.24) and a maximum value of 0.98.

For comment-induced engagement, the mean value of $\alpha_{\text{2}}^{i}\;$is −0.412, suggesting that comments, on average, negatively affect retweet diffusion. However, the observed variation indicates that this effect is heterogeneous across posts, with comments potentially exerting stronger or weaker influence depending on the context. The decay parameter $\beta_{\text{2}}^{i}$, with a mean of 0.667, indicates that comment effects also diminish over time, following a similar but more persistent decay pattern compared to retweets.

For cross-cascade effects, the parameter $\alpha_{\text{3}}j^{i}\;$ has a mean of −0.193, suggesting that, on average, parallel information streams tend to exert a suppressive effect on each other, consistent with competition for user attention. However, the wide range of values indicates that cross-cascade interactions can also be reinforcing in certain cases. The corresponding decay parameter $\beta_{\text{3}}j^{i}$, with a mean of 0.859, implies that cross-cascade effects persist longer than self-excitation effects before dissipating. Because the comment-induced and cross-cascade parameters are intentionally unrestricted in sign, intensity non-negativity is determined by the total fitted intensity after all model components are combined, rather than by the α₁ / β₁ branching-stability condition alone. We therefore conduct an ex post intensity validation by reconstructing λᵢ(t) for each post using the baseline intensity, retweet self-excitation, comment effect, and cross-cascade effect, evaluated at observed event times and on a regular grid within the 10-hour observation window. The fitted total intensity remains non-negative throughout the observation window for approximately 99.7% of posts. The remaining 87 posts, approximately 0.3% of the estimated sample, show numerical violations and are excluded from the final estimation sample.

**Fig 7:**
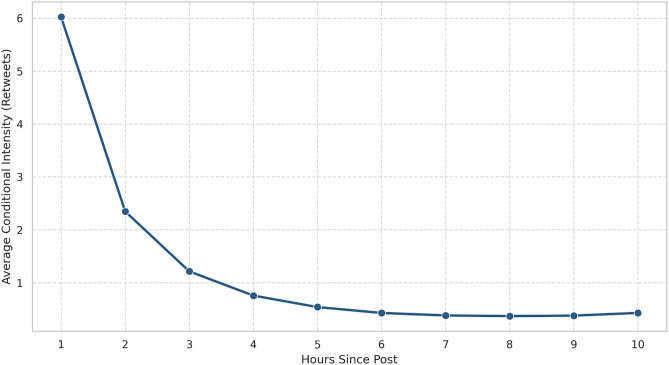
Average Hawkes intensity over time. The figure shows the estimated conditional intensity of retweets, reflecting the instantaneous rate of engagement over the 10-hour period.

Fig 7 illustrates the temporal evolution of the average Hawkes intensity across all posts. Initially, the intensity declines sharply, reflecting the rapid decay of user engagement shortly after a post is published. This pattern is consistent with the self-exciting nature of social media interactions, where engagement spikes immediately following a post but gradually diminishes over time.

Between two and six hours after posting, the intensity decline slows, suggesting a stabilization phase in which engagement continues at a lower, more stable rate. Notably, after approximately six hours, the average intensity reaches its lowest point, indicating that most engagement activity has subsided. However, a slight upward trend emerges beyond this point, suggesting that secondary interactions, such as follow-up comments or renewed interest, contribute to a minor resurgence in engagement.

This temporal pattern highlights the importance of the initial engagement window in shaping diffusion dynamics. The sharp early decline emphasizes the need for content creators to maximize visibility during the first few hours after posts. Additionally, the slight late-stage increase in intensity suggests opportunities for re-engagement strategies, such as reposting or encouraging discussions to sustain audience interest.

### Parameter robustness and goodness-of-fit

The accuracy of these parameters is crucial as they directly influence the model’s ability to capture the temporal dynamics and interaction effects of information diffusion. Moreover, ensuring that the estimated parameters adhere to expected statistical patterns provides a solid foundation for using these parameters as prior knowledge in data-driven prediction models, such as deep learning, thereby enhancing both their interpretability and predictive reliability.

To assess parameter robustness and theoretical alignment, we employ Kernel Density Estimation (KDE) to examine their empirical distributions. While KDE-based assessments do not directly quantify goodness-of-fit, they serve as an essential diagnostic tool for identifying potential estimation biases or data-driven anomalies. The KDE plot allows us to assess key statistical properties, such as central tendency, dispersion, skewness, and multimodality. By analyzing parameters like dynamic baseline intensity ($\mu_{\text{0}}$ and γ), self-excitation ($\alpha_{self}$), and cross-excitation ($\alpha_{cross}$), we evaluate whether the estimated values accurately reflect observed diffusion patterns. As shown in S1–S8 Figs, the results reveal multimodal distributions for certain parameters, suggesting heterogeneity in user engagement and content virality. Additionally, the KDE distributions closely match empirical data, confirming the robustness of the parameter estimation process.

To further complement this diagnostic evaluation, we also conduct a formal goodness-of-fit test for the point process model using the residual point method, based on Papangelou’s random time-change theorem. The cumulative intensity process is expressed as:


$$\begin{array}{c} \Lambda (t)=\int_{\text{0}}^{t}\lambda (s)ds \end{array}$$

The corresponding transformed counting process is:
$N\left(\Lambda^{-\text{1}}(t)\right)$.

If the model accurately captures the characteristics of retweet data on Sina Weibo, the estimated arrival times of retweet events t=1,…,N(T) for a given piece of content i should result in inter-arrival residuals:


$$\begin{array}{c} \left\{\Lambda \left({\hat{\tau }}_{\text{1}}\right),\Lambda \left({\hat{\tau }}_{\text{2}}\right),\Lambda \left({\hat{\tau }}_{\text{3}}\right),\ldots \right\}=\left\{t_{\text{1}}^{*},t_{\text{2}}^{*}-t_{\text{1}}^{*},t_{\text{3}}^{*}-t_{\text{2}}^{*},\ldots \right\} \end{array}$$

These residuals are expected to follow an exponential distribution with a unit rate:

$\tau_{i}\sim \text{exp}(\text{1})$

**Fig 8:**
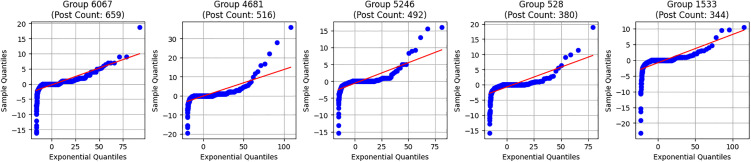
Q–Q plots of model residuals for the five largest topic groups. The plots compare empirical residual quantiles with theoretical values to assess model fit.

To provide a systematic evaluation, we compute Kolmogorov–Smirnov (KS) statistics for the rescaled inter-event times across all posts and report the results in Table 5, stratified by cascade size. Given the large sample size, generating Q-Q plots for all groups is impractical. Therefore, we select the five topics with the largest number of posts, ensuring a broad and diverse representation of the data. Q-Q plots for these groups are presented in Fig 8, offering a visual assessment of the model’s fit. For most selected groups, the data points align well with the theoretical quantiles, supporting the validity of the model’s assumptions. In particular, the majority of groups exhibit a strong fit to the exponential distribution, as evidenced by the close alignment of the data points with the theoretical line.

**Table 5:** Goodness-of-fit statistics based on KS tests of time-rescaled residuals.

Cascade Scale	Number of Posts (N)	Mean KS Statistic	Mean p-value	Pass Rate (p > 0.01)
Small (<10)	236	0.513	0.148	93.22%
Medium (10–20)	1,170	0.651	0.046	41.79%
Large (>20)	1,320	0.797	0.012	13.71%

As expected, some topics display deviations in the tail, particularly for posts with extreme engagement levels. To further investigate this pattern, we conduct additional analyses on subsamples where heavy-tailed behavior is most likely to arise, including (i) the top 5% most viral posts (retweet volume above the 95th percentile) and (ii) the largest topic clusters. The results indicate that tail deviations are systematically more pronounced among high-virality posts, confirming that heavy-tailed engagement is concentrated in extreme diffusion cases. However, despite these deviations, the exponential decay specification continues to provide a strong overall fit across the majority of observations.

This finding suggests that heavy-tailed behavior primarily manifests in the extreme tail of a relatively small subset of posts, rather than dominating the overall temporal dynamics of engagement. In our setting, most diffusion activity is driven by rapid early-stage interactions, which are effectively captured by the exponential decay kernel. To further assess kernel adequacy, we conduct a robustness check by comparing the exponential kernel with a truncated power-law specification. We estimated both models on two critical subsamples most likely to exhibit long-tail deviations: (1) the five largest topic groups, and (2) the top 5% highest-virality posts (by retweet volume). Contrary to theoretical expectations regarding heavy-tailed distributions, the exponential kernel consistently outperformed the power-law alternative across both subsamples. For the five largest topic groups, the exponential kernel achieved a substantially lower AIC (8,467.25) compared to the power-law kernel (15,766.91). Similarly, for the top 5% viral posts, the exponential kernel exhibited superior fit (AIC = −168,161.03) versus the power-law kernel (AIC = −126,212.78).

The substantially lower KS pass rate among large cascades indicates that the exponential Hawkes specification fits ordinary and medium-scale diffusion processes more reliably than highly viral cascades. Because large cascades are more likely to involve delayed bursts, external shocks, influencer reposts, or algorithmic re-ranking, their residual processes deviate more strongly from the unit-rate exponential benchmark. Accordingly, Hawkes parameters estimated for this subgroup should be interpreted cautiously. They remain useful as structured summaries of early-stage excitation and competition, but they may not fully capture the long-tail dynamics of extreme diffusion events.

### Comment sentiment analysis

To more rigorously assess the comment suppression effect, it is necessary to account for the sentiment polarity of user comments, since the observed reduction in information diffusion could otherwise be confounded by the presence of negative or controversial discourse. To disentangle the effect of comment volume from that of comment sentiment, we employ a fine-tuned BERT model (BERT-base-Chinese, optimized for social media corpora) to estimate sentiment polarity scores for the comments associated with each focal post. Specifically, for each time interval, we construct a dynamic sentiment variable defined as the proportion of negative comments among all comments accumulated for a given post up to that point. This sentiment measure is then incorporated into the model as a time-varying covariate.

Our modeling framework separates behavioral dynamics from semantic information. The Hawkes process captures the temporal and interactive patterns of user behavior without directly modeling textual semantics, whereas the Transformer prediction layer integrates the extracted sentiment information together with historical features to predict future retweet volume. The results show that the negative association between comment intensity and subsequent retweet activity remains highly robust after controlling for comment sentiment. In addition, sentiment itself plays a significant moderating role. We find a significantly negative relationship between the proportion of negative comments and future retweet volume (coefficient = −0.0412, p < 0.05). Substantively, a one-standard-deviation increase in the share of negative comments is associated with a reduction of approximately 0.0412 retweets per hour. Relative to the baseline mean of 1.2476 retweets per hour, this corresponds to a 3.30% decrease in future retweet increments. These findings suggest that a higher prevalence of negative comments further inhibits users’ willingness to propagate the focal message.

## Transformer-based model with prior knowledge

We incorporate Hawkes parameters as prior knowledge into a Transformer-based model including baseline intensity ($\mu_{\text{0}}$), self-excitation engagement parameters ($\alpha_{self},\beta_{self}$), cross-excitation engagement parameters ($\alpha_{cross},\beta_{cross}$), comment-induced engagement parameters ($\alpha_{comment},\beta_{comment}$), and like-induced engagement parameter ($\gamma_{like}$). The dynamic features from Sina Weibo, used as observed data, include metrics such as cumulative retweets, comments, active users, and comment-to-retweet ratios.

The eight Hawkes parameters estimated per post (*μ*₀, α_self, β_self, α_cross, β_cross, α_comment, β_comment, γ) are concatenated with the time-varying engagement features (hourly retweets, hourly comments, active users and sentiment) to form a 12-dimensional input vector at each time step, before being fed into the Transformer encoder. Because Hawkes parameters are post-level constants, they are repeated (broadcasted) across all 10 time steps for each post, thereby serving as static features aligned with the temporal sequence. This design allows the Hawkes parameters to act as structural priors that condition the attention mechanism, enabling the Transformer to incorporate information about self-excitation, cross-cascade interactions, and decay dynamics when modeling temporal dependencies in engagement behavior. In this way, the model combines time-varying signals with interpretable structural parameters within a unified representation.

To ensure numerical stability, we impute missing values using column-wise means and standardize all features. The dataset is then split using a temporal strategy rather than a random partition. Specifically, for each post’s 10-hour observation window, the first 80% of the timeline (first 8 hours) is used for training (including validation), while the remaining 20% (last 2 hours) is reserved for testing. The validation set is drawn from the training period and is used for hyperparameter tuning and model selection, while the testing set is used for final evaluation. Formally, let $X_{t}$ represent the feature vector at time t, which includes both Hawkes parameters and observed engagement features. The objective is to learn a function $f_{\theta }$ parameterized by the Transformer model that maps the input sequence to future engagement levels:

${\hat{y}}_{t+h}=f_{\theta }(X_{t},X_{t-\text{1}},\ldots .,X_{t-w})$

where w = 10 represents the observation window size (10 hourly time steps), and h = 1 represents the forecasting horizon (one-step-ahead prediction). The model is trained using a mean squared error (MSE) loss function:


$$\begin{array}{c} L=\;\frac{\text{1}}{N}\sum_{i=\text{1}}^{N}{\left(y_{i}-{\hat{y}}_{i}\right)}^{\text{2}} \end{array}$$

where $y_{i}\;$is the actual engagement count and ${\hat{y}}_{i}\;$is the predicted value.

In addition to the training settings described above, we specify the Transformer architecture as shown in Table 6. The input dimension is 12, corresponding to eight Hawkes parameters and four additional behavioral features (comments, retweet, active users, and sentiment). We use two attention heads and stack two encoder layers. The feed-forward network has a hidden dimension of 32, and the output dimension is 2, corresponding to the prediction length. A standard dropout rate of 0.1 is applied following the default Transformer configuration.

**Table 6:** Transformer hyperparameters.

Category	Parameter	Value
Architecture	Input dimension	12
	Number of attention heads	2
	Number of encoder layers	2
	Feed-forward dimension	32
	Output dimension	2
	Dropout rate	0.1
Training	Batch size	512
	Number of epochs	100
	Loss function	MSE
Optimization	Optimizer	Adam
	Learning rate	0.001
	Learning rate scheduler	LinearLR
	Gradient clipping	max_norm = 0.5
	Mixed precision	Enabled

For training and optimization, we use the Adam optimizer with a learning rate of 0.001 and a batch size of 512. A linear learning rate scheduler (LinearLR) is applied to decay the learning rate over the training process. The model is trained for 100 epochs using mean squared error (MSE) as the loss function. Gradient clipping is implemented with a maximum norm of 0.5 to ensure stable training. Mixed-precision training is enabled to improve computational efficiency. After training, we evaluate the model using five performance metrics: mean squared error (MSE), mean absolute error (MAE), root mean squared error (RMSE), R^2^, and mean absolute percentage error (MAPE). The results, summarized in Table 7, demonstrate strong predictive accuracy across both tasks, characterized by low error rates and robust goodness-of-fit.

**Table 7:** Summary of evaluation metrics.

Metric	Retweet	Active user
MSE	0.6857	1.4546
MAE	0.3090	0.5992
R^2^	0.1397	0.1465
RMSE	0.8281	1.2061
MAPE	65.5080%	51.4732%

For the retweet prediction task, the model achieves an MSE of 0.6857, RMSE of 0.8281, and a MAPE of 65.51%, indicating strong alignment between predicted and actual engagement levels. The R^2^ value of 0.1397 further confirms that the model captures the variance in retweet dynamics effectively, showcasing the significant role of Hawkes parameters in learning temporal dependencies. While retweet counts offer some indication of a topic’s reach, they may not accurately reflect the true extent of information diffusion, especially when engagement is driven by a small number of highly active users. Therefore, we introduce active users, defined as the number of unique users engaging with a topic, as an additional prediction metric to capture the breadth of audience participation. This measure complements retweet-based metrics by reflecting how widely attention is distributed across users, rather than how frequently content is reshared.For the retweet prediction task, the model achieves an MSE of 0.6857, RMSE of 0.8281, and a MAPE of 65.51%, indicating strong alignment between predicted and actual engagement levels. The R^2^ value of 0.1397 further confirms that the model captures the variance in retweet dynamics effectively, showcasing the significant role of Hawkes parameters in learning temporal dependencies. While retweet counts offer some indication of a topic’s reach, they may not accurately reflect the true extent of information diffusion, especially when engagement is driven by a small number of highly active users. Therefore, we introduce active users, defined as the number of unique users engaging with a topic, as an additional prediction metric to capture the breadth of audience participation. This measure complements retweet-based metrics by reflecting how widely attention is distributed across users, rather than how frequently content is reshared.

For active user prediction, the model achieves an MSE of 1.4546 and an RMSE of 1.2061, demonstrating performance comparable to the retweet prediction task. The high $R^{\text{2}}$ value of 0.1465 indicates a strong fit, suggesting that the model effectively captures patterns in user participation dynamics. However, the higher MAPE of 51.47% reflects greater variability in active user participation compared to retweet behavior, consistent with the more heterogeneous and dispersed nature of audience engagement across users.

Overall, these results demonstrate the effectiveness of the Hawkes--Transformer framework for forecasting social media engagement. By using Hawkes process parameters as prior knowledge, our Transformer-based deep learning model accurately captures the dynamics of information diffusion and demonstrates its potential for real-time engagement prediction in dynamic online environments. In this study, we conduct an ablation analysis to assess the contribution of the Hawkes-estimated parameters in improving the predictive performance and convergence speed of the model. Specifically, we investigate the impact of including Hawkes-estimated parameters by comparing the full Hawkes--Transformer model with a variant that removes the Hawkes-estimated parameters from the Transformer model.

**Fig 9:**
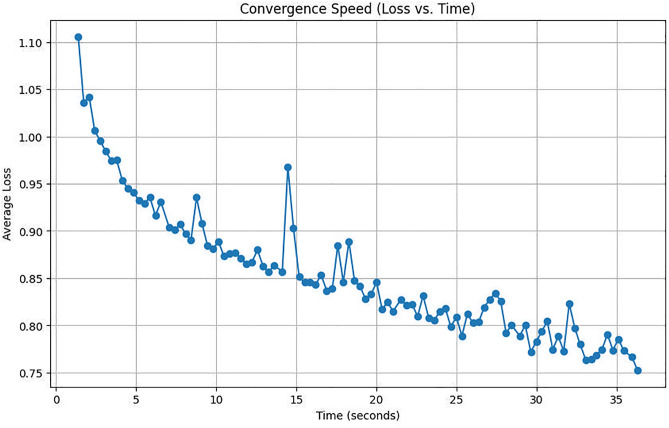
Training loss over time for retweet prediction using the Hawkes–Transformer model.

**Fig 10:**
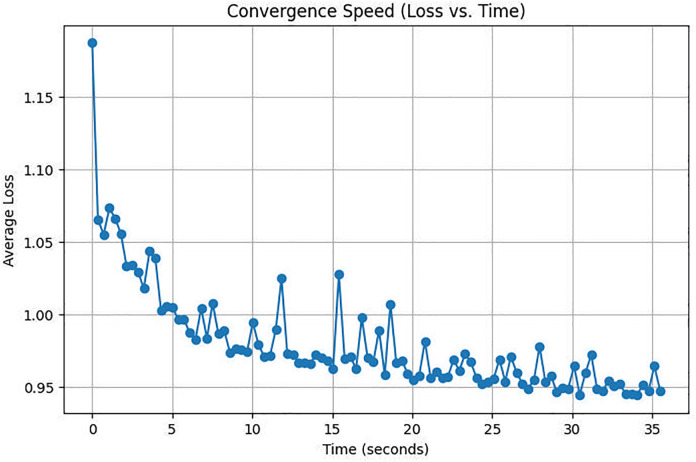
Training loss over time for retweet prediction using the baseline Transformer model.

**Fig 11:**
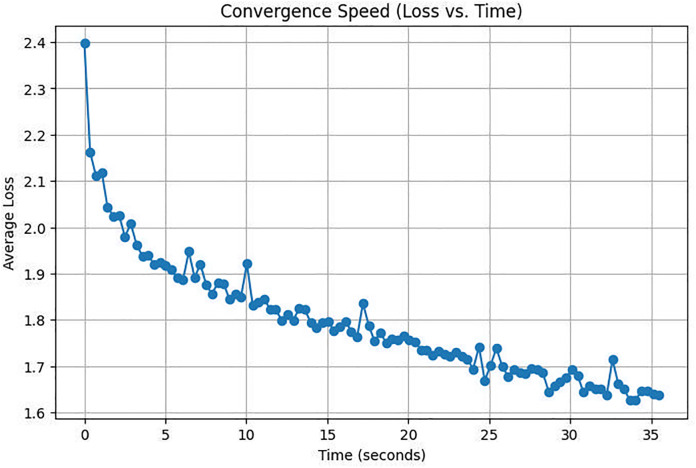
Training loss over time for active user prediction using the Hawkes–Transformer model.

**Fig 12:**
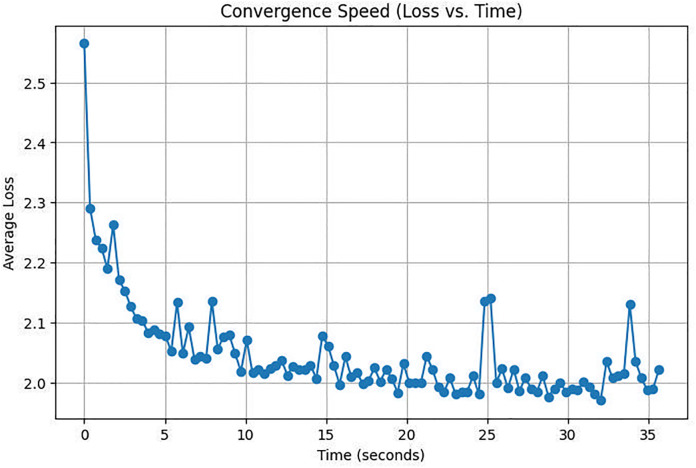
Training loss over time for active user prediction using the baseline Transformer model.

Table 8 and Figs 9–12 summarize the experimental results. The findings show that incorporating the Hawkes-estimated parameters, alongside historical data like retweet counts, comment counts, and active user numbers, leads to substantial improvements in prediction performance metrics, such as lower MSE, MAE, and higher R^2^. Furthermore, the inclusion of the Hawkes parameters is associated with more efficient convergence behavior, as the model reaches a lower loss level compared to a baseline deep learning model without these parameters. These results demonstrate that combining theory-driven statistical models, such as the Hawkes model, with data-driven deep learning models is a promising approach. The Hawkes--Transformer model shows that deep learning can effectively leverage Hawkes-estimated parameters as prior knowledge, thereby enhancing both the interpretability and predictive power of the model.

**Table 8:** Ablation results: Hawkes-Transformer vs. Transformer without Hawkes parameters.

Model	Task	MSE	MAE	R²	RMSE	MAPE (%)
Hawkes–Transformer	Retweet	0.6857	0.3090	0.1397	0.8281	65.5080
Transformer (no Hawkes)	Retweet	0.7253	0.3495	0.0914	0.8517	69.6156
Hawkes–Transformer	Active User	1.4546	0.5992	0.1465	1.2061	51.4732
Transformer (no Hawkes)	Active User	1.4718	0.6483	0.1361	1.2132	51.0885

To further assess whether the predictive contribution of Hawkes-estimated parameters is robust to cascade-size heterogeneity, we repeat the ablation comparison across cascade-size subgroups. Specifically, we exclude posts with more than 20 retweets and re-estimate the forecasting models on the small- and medium-cascade sample; we also analyze the large-cascade subgroup separately. For both checks, we preserve the same temporal split used in the main analysis, the same input construction, and the same evaluation metrics. The results are reported in Table 9.

**Table 9:** Robustness results by cascade-size subgroup.

Sample	Model	MSE	MAE	R²	RMSE	MAPE (%)
Excl. large	Hawkes–Transformer	0.3802	0.2723	0.0575	0.6166	68.8145
Excl. large	Transformer	0.4006	0.3187	0.0084	0.6330	75.8929
Large only	Hawkes–Transformer	1.6449	0.4066	0.1847	1.2825	64.1773
Large only	Transformer	1.7336	0.4536	0.1352	1.3166	67.4369

*Note:* “Excl. large” refers to the sample excluding posts with more than 20 retweets; “Large only” refers to posts with more than 20 retweets.

The subgroup results show that the Hawkes--Transformer retains its advantage over the Transformer without Hawkes parameters in both subsamples. In the sample excluding large cascades, the Hawkes--Transformer achieves lower MSE, MAE, RMSE, and MAPE, together with a higher R^2^. The same pattern also holds in the large-cascade-only sample. Although large cascades are more difficult to predict in absolute scale, the Hawkes--Transformer achieves a higher R² in this subgroup than in the sample excluding large cascades, suggesting that the hybrid model still captures meaningful variation in large-cascade diffusion patterns.

After examining the ablation results and the convergence patterns, we further benchmark the proposed Hawkes–Transformer against a broader set of competing forecasting models. Table 10 reports the comparison with standard sequence models, a neural Hawkes baseline, and a standalone Hawkes-based forecasting baseline. This comparison allows us to distinguish two sources of performance: the value of incorporating Hawkes-derived dynamic features into the Transformer, and the incremental contribution of the Transformer layer beyond the analytical Hawkes specification alone.

**Table 10:** Comparison of competitive models on incremental prediction tasks.

Panel A. Retweet Prediction						
**Model**	**MSE**	**MAE**	**RMSE**	**R²**	**MAPE (%)**	**SMAPE (%)**
Hawkes–Transformer	0.6857	0.3090	0.8281	0.1397	65.5080	189.514
Transformer	0.7253	0.3495	0.8517	0.0914	69.6156	190.069
Pure LSTM	0.7280	0.3331	0.8533	0.0875	66.4138	190.515
KAWES (Neural Hawkes)	0.7248	0.3112	0.8513	0.0868	62.8249	188.467
Hawkes-only baseline	12.2659	1.5766	3.5022	−14.4109	59.0335	184.656
Panel B. Active User Prediction						
**Model**	**MSE**	**MAE**	**RMSE**	**R²**	**MAPE (%)**	**SMAPE (%)**
Hawkes–Transformer	1.4546	0.5992	1.2061	0.1465	51.4732	172.052
Transformer	1.4718	0.6483	1.2132	0.1361	51.0885	171.841
Pure LSTM	1.5028	0.6408	1.2259	0.1192	50.1446	171.984
KAWES (Neural Hawkes)	1.6211	0.6147	1.2732	0.0501	52.1264	172.048

*Note:* The Hawkes-only baseline is analytically defined for retweet intensity only and is therefore reported in Panel A to quantify the incremental value of the Transformer layer. It is omitted from Panel B because there is no direct intensity counterpart for active user arrivals.

For retweet prediction in Panel A, the Hawkes–Transformer achieves the strongest overall performance on the main scale-dependent and variance-based metrics, with the lowest MSE and RMSE and the highest R-squared. Its advantage over the Transformer without Hawkes inputs is consistent with the ablation results in Table 8, indicating that the Hawkes-estimated parameters provide useful structured information about early diffusion dynamics. The comparison with LSTM and KAWES further suggests that neither standard sequence modeling nor an end-to-end neural point process alone fully captures the combination of self-excitation, cross-cascade competition, and contextual variation present in the data.

The Hawkes-only baseline provides a more direct test of the incremental contribution of the Transformer layer. This baseline performs substantially worse than the neural models on scale-dependent and variance-based metrics, with an MSE of 12.2659 and an R-squared of −14.4109, compared with an MSE of 0.6857 and an R-squared of 0.1397 for the Hawkes–Transformer. This contrast does not imply that the Hawkes component is uninformative. Rather, it indicates that the analytical Hawkes intensity alone is insufficient for short-horizon micro-level forecasting when early retweet histories are sparse and heterogeneous. The Transformer layer therefore plays a calibration and feature-integration role: it uses the structured Hawkes intensity together with dynamic contextual features to model nonlinear interactions among retweets, comments, sentiment, and contemporaneous user activity.

For active-user prediction in Panel B, the Hawkes–Transformer also remains competitive across the main metrics. We do not report a Hawkes-only baseline for this task because the analytical Hawkes specification is defined for retweet intensity and does not provide a direct intensity counterpart for active user arrivals. The Panel B results therefore serve mainly to evaluate whether the hybrid representation remains useful for a related engagement outcome beyond retweet counts.

Finally, the percentage-based metrics in Table 10 should be interpreted cautiously. Because the forecasting targets are hourly incremental counts, the data contain substantial zero-inflation, with many post-hour observations having zero new retweets. In this sparse-count setting, MAPE and SMAPE are highly sensitive to observations with zero or near-zero denominators and may overstate the substantive magnitude of prediction error. We therefore retain MAPE and SMAPE as secondary diagnostic metrics and for comparability with prior studies, but we base the main model comparisons on MSE, MAE, RMSE, and R-squared, which are not affected by zero denominators and more directly reflect the model’s ability to capture nonzero variation in incremental engagement.

## Discussion and implications

Our results reveal distinct and sometimes opposing influences of different engagement activities on content diffusion. Retweets exhibit strong self-exciting dynamics, confirming their role as the primary driver of information spread on social platforms. This amplification effect stems not only from increased visibility but also from social proof mechanisms, where early retweet activity signals popularity and triggers further diffusion. In contrast, likes exert a moderate reinforcing influence on retweet activity. This effect likely operates through confirmation heuristics: users perceive liked content as trustworthy or valuable, which increases their willingness to propagate it. However, because likes do not redistribute content to new networks, their amplification is indirect and limited in scale.

Comments, however, show a moderate suppressive effect on retweet diffusion. This suppression can be understood through three mechanisms: (1) a substitution effect, where users choose to comment instead of retweeting, allocating their attention and effort toward conversation rather than content spread; (2) sentiment moderation, where negative or controversial comments discourage others from associating with the content; and (3) engagement fragmentation, in which excessive commenting absorbs user attention and reduces the momentum of further sharing. These findings challenge the common perception that all engagement signals enhance visibility and underscore the need to distinguish between engagement types.

Furthermore, we find that cross-cascade competition significantly suppresses content diffusion. When multiple posts under the same topic compete for user attention, diffusion becomes fragmented. Algorithmic ranking may exacerbate this by promoting some posts over others, creating a “winner-takes-most” environment. This competition is particularly evident when cascades are temporally close, confirming the temporal sensitivity of attention allocation in social media ecosystems.

### Theoretical contributions

This study contributes to the broader literature on digital communication, information diffusion, and socio-technical change by introducing a comprehensive framework that unifies multivariate self-excitation, cross-behavioral interaction, and cascade competition within a single modeling and forecasting paradigm. It advances theoretical understanding of how human engagement and algorithmic mediation jointly shape the evolution of collective attention in digital environments.

First, the study extends prior research that treats retweets, comments, and likes as independent behaviors [8,9] by modeling the interdependent and asymmetric dynamics across engagement types. We show that some interactions generate reinforcing effects (e.g., likes amplifying retweet diffusion), whereas others exhibit substitution or suppressive effects (e.g., comments moderating retweet cascades). These findings uncover a non-additive structure of social interaction, enriching theoretical perspectives on how emotional, informational, and social signals co-evolve in digital diffusion processes.

Second, we address the underexplored dynamics of cross-cascade interaction by modeling how parallel information flows under the same trending topic compete or reinforce one another. While previous research typically assumes cascades to be independent, our results reveal the competitive and fragmented nature of attention in algorithmically curated ecosystems. This perspective extends theories of attention scarcity and information competition, highlighting how digital environments increasingly mediate collective cognition and public discourse.

Third, we bridge theory-driven modeling with data-driven forecasting by embedding Hawkes-estimated temporal dependencies into a Transformer-based architecture. This hybrid integration demonstrates how behaviorally grounded statistical structures can serve as prior knowledge to inform deep learning, providing a transparent and interpretable analytical lens for complex social phenomena. It contributes to the emerging discourse on hybrid analytical intelligence, which reconciles statistical rigor with machine learning scalability for studying socio-technical systems.

Together, these contributions advance theoretical understanding of the interplay between engagement behaviors, information cascades, and algorithmic attention mechanisms, offering foresight into how human–algorithm interactions will continue to shape information visibility and diffusion in the evolving digital society.

### Practical implications

Our findings provide actionable insights for platform designers, digital marketers, and social media strategists seeking to optimize engagement outcomes and manage content visibility in competitive digital environments.

First, the strong self-excitation effect of retweets suggests that early-stage engagement is critical. Platforms and marketers should monitor early retweet trajectories to predict organic virality and decide when to trigger additional promotion (e.g., influencer reposts, paid boosts). Algorithmic prioritization of content could incorporate real-time estimates of excitation intensity to dynamically allocate feed exposure.

Second, the nuanced role of comments—sometimes reinforcing, often suppressive—suggests that not all engagement is beneficial for diffusion. Firms and platforms may consider distinguishing engagement for feedback (comments) from engagement for amplification (retweets). For example, algorithmic systems could down-weight controversial or high-negativity comment threads when predicting shareability or allocating visibility.

Third, the evidence of cross-cascade suppression implies that timing content releases to avoid competition with similar posts may increase visibility. Platforms may benefit from content-staggering algorithms that manage temporal spacing among related posts to mitigate attention cannibalization within trending topics.

Fourth, the hybrid Hawkes--Transformer framework demonstrates that combining structural engagement patterns with real-time behavioral data enables more accurate and adaptive prediction of content success. This capability can support real-time dashboard tools for social media teams to adjust campaign timing, promotion intensity, and content formats based on predicted diffusion curves.

### Limitations and future research

Despite its contributions, this study has several limitations that suggest avenues for future work. First, the model is grounded in behavioral event patterns but does not incorporate the semantic and cognitive dimensions of content. While we capture “how” users interact (retweet, comment, like), we do not account for “why” they interact, such as message framing, emotional tone, or cultural alignment. Future research could integrate natural language features and affective content analysis to enrich the explanatory depth of engagement mechanisms.

Second, the Hawkes--Transformer framework is developed and evaluated on Sina Weibo, a platform with specific affordances that may shape diffusion dynamics including the Hot Search ranking algorithm, Weibo’s repost-centric architecture, and China’s distinct social media user culture. The competitive cascade dynamics identified here (predominant negative cross-cascade effects within trending topics) may manifest differently on platforms with different recommendation algorithms, content moderation policies, or user interaction norms. Replication studies on Twitter/X, Reddit, or TikTok data would be needed to assess the cross-platform generalizability of our findings.

Third, another limitation relates to sample selection. Our dataset consists exclusively of posts that appeared on Sina Weibo’s Hot Search List at least once during the study period. As such, the analysis focuses on high-visibility and trending content, which may not fully represent long-tail or non-trending posts. Engagement dynamics in less visible content may differ in terms of user participation patterns, diffusion speed, and cross-cascade interactions. Therefore, the generalizability of our findings beyond trending topics is limited, and future research should validate the proposed framework using broader and more representative samples of social media content.

Fourth, the model assumes an exponential decay structure for the influence of past events, which may not fully capture long-tail or bursty diffusion patterns in social media environments. Our goodness-of-fit analysis suggests that while the exponential specification provides a strong fit for the majority of cascades, deviations are more likely to occur in a small subset of highly viral posts, where heavy-tailed engagement becomes more prominent. In such cases, more flexible kernels (e.g., power-law or Weibull) may better capture tail behavior. However, given that most diffusion activity in our setting is driven by early-stage dynamics, the exponential kernel remains an effective approximation for the dominant process. Future research could explore adaptive kernel specifications to better capture both early-stage decay and extreme tail behavior.

Finally, our hybrid framework emphasizes statistical structure and prediction, but future work could extend it to decision-making contexts, such as adaptive content scheduling, real-time engagement interventions, or A/B testing of content strategies based on predicted engagement and diffusion dynamics.

## Supporting information

S1 Fig KDE and histogram of the baseline intensity parameter (μ_0_). This figure shows the empirical distribution and kernel density estimate of μ_0_ across posts. The distribution is right-skewed, indicating that most posts exhibit low baseline activity, while a small number have substantially higher intrinsic popularity.
(TIF)

S2 Fig KDE and histogram of the like effect parameter (γ_like). The distribution of γ_like across posts exhibits notable dispersion, indicating that the contribution of likes to baseline engagement varies substantially across content. This heterogeneity suggests that likes may play different roles in reinforcing user attention depending on post characteristics.
(TIF)

S3 Fig KDE and histogram of the self-excitation parameter (α_self). The distribution of α_self exhibits considerable dispersion, indicating that the strength of self-excitation varies substantially across posts. This suggests that the reinforcing effect of retweets on subsequent retweet activity is highly heterogeneous, with some posts generating strong cascading dynamics while others exhibit limited propagation.
(TIF)

S4 Fig KDE and histogram of the decay parameter (β_self). This figure shows the empirical distribution and kernel density estimate of β_self across posts. The distribution indicates variation in the decay rate of self-excitation, suggesting that the influence of past retweets diminishes at different speeds across posts.
(TIF)

S5 Fig KDE and histogram of the cross-excitation parameter from comments (α_comment). This figure shows the empirical distribution and kernel density estimate of α_comment across posts. The distribution indicates heterogeneity in how comments influence subsequent retweet activity, suggesting that the cross-excitation effect varies substantially across content.
(TIF)

S6 Fig KDE and histogram of the decay parameter for comment-induced effects (β_comment). This figure shows the empirical distribution and kernel density estimate of β_comment across posts. The distribution indicates variation in the rate at which the influence of comments on subsequent retweet activity decays over time.
(TIF)

S7 Fig KDE and histogram of the cross-cascade excitation parameter (α_cross). This figure shows the empirical distribution and kernel density estimate of α_cross across posts. The distribution indicates heterogeneity in the extent to which activity from other cascades influences retweet dynamics, suggesting that cross-cascade interactions vary substantially across content.
(TIF)

S8 Fig KDE and histogram of the decay parameter for cross-cascade effects (β_cross). This figure shows the empirical distribution and kernel density estimate of β_cross across posts. The distribution indicates variation in how quickly the influence of parallel information cascades on retweet activity decays over time.
(TIF)

S1 Data Dataset Description and Aggregated Data for Replication. This dataset contains aggregated engagement information (e.g., retweets, comments, and active user counts) used in the study. The data were collected from publicly accessible Sina Weibo content via API-based procedures in accordance with the platform’s terms of service, and do not include any personally identifiable information.
(ZIP)
