# Supplementary figures and images for: Forecasting user engagement and competing cascades in social media diffusion: A Hawkes-Transformer approach
Source: PLoS One. 2026 Jul 24;21(7):e0354472. doi: 10.1371/journal.pone.0354472 (PMC13399361; doi:10.1371/journal.pone.0354472)

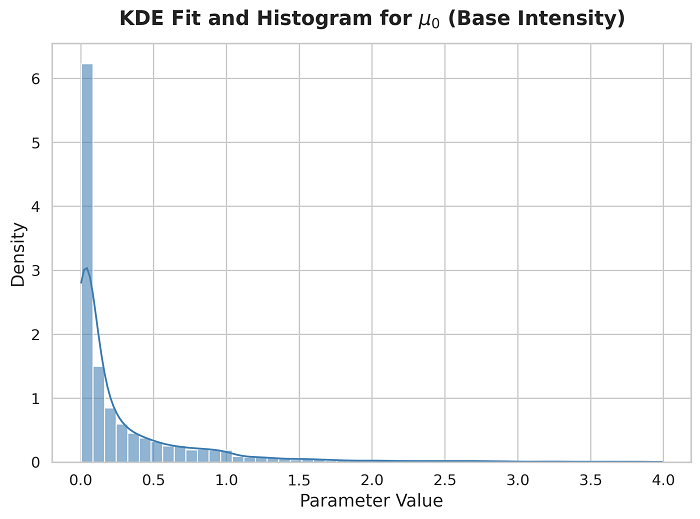

Supplement: S1 Fig — This figure shows the empirical distribution and kernel density estimate of μ0 across posts. The distribution is right-skewed, indicating that most posts exhibit low baseline activity, while a small number have substantially higher intrinsic popularity. (TIF) [file pone.0354472.s001.tif]

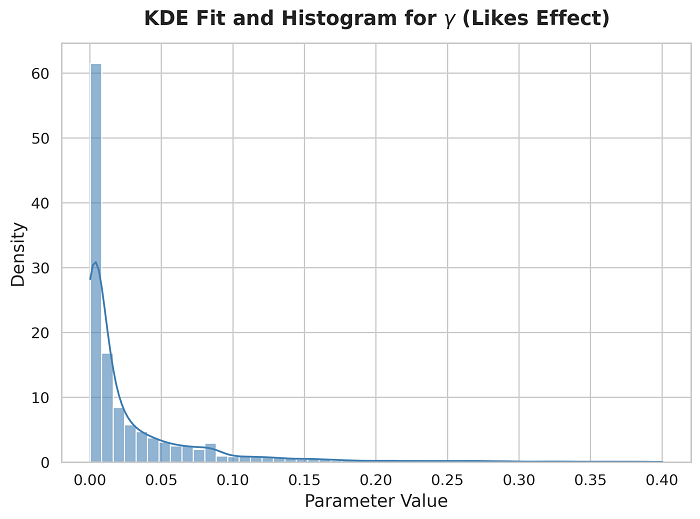

Supplement: S2 Fig — The distribution of γ_like across posts exhibits notable dispersion, indicating that the contribution of likes to baseline engagement varies substantially across content. This heterogeneity suggests that likes may play different roles in reinforcing user attention depending on post characteristics. (TIF) [file pone.0354472.s002.tif]

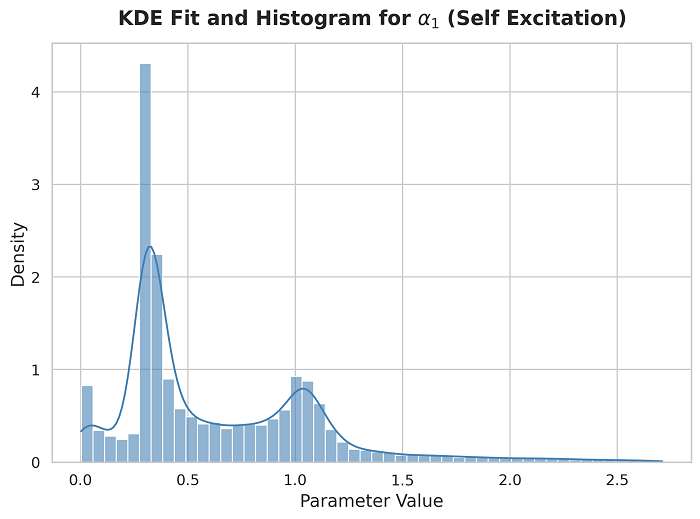

Supplement: S3 Fig — The distribution of α_self exhibits considerable dispersion, indicating that the strength of self-excitation varies substantially across posts. This suggests that the reinforcing effect of retweets on subsequent retweet activity is highly heterogeneous, with some posts generating strong cascading dynamics while others exhibit limited propagation. (TIF) [file pone.0354472.s003.tif]

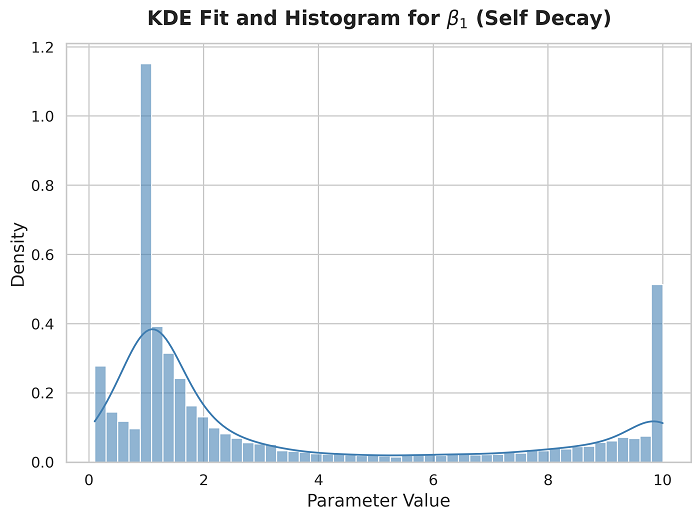

Supplement: S4 Fig — This figure shows the empirical distribution and kernel density estimate of β_self across posts. The distribution indicates variation in the decay rate of self-excitation, suggesting that the influence of past retweets diminishes at different speeds across posts. (TIF) [file pone.0354472.s004.tif]

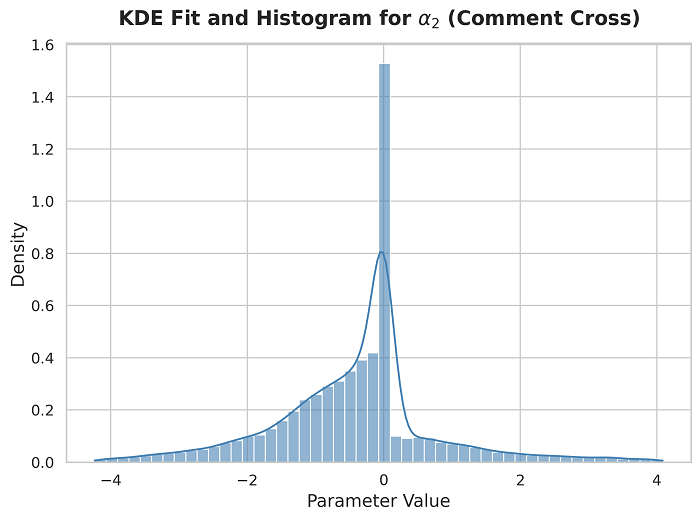

Supplement: S5 Fig — This figure shows the empirical distribution and kernel density estimate of α_comment across posts. The distribution indicates heterogeneity in how comments influence subsequent retweet activity, suggesting that the cross-excitation effect varies substantially across content. (TIF) [file pone.0354472.s005.tif]

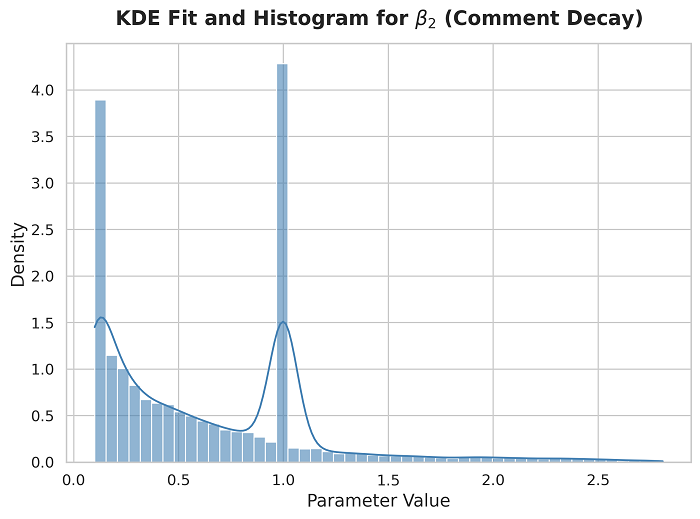

Supplement: S6 Fig — This figure shows the empirical distribution and kernel density estimate of β_comment across posts. The distribution indicates variation in the rate at which the influence of comments on subsequent retweet activity decays over time. (TIF) [file pone.0354472.s006.tif]

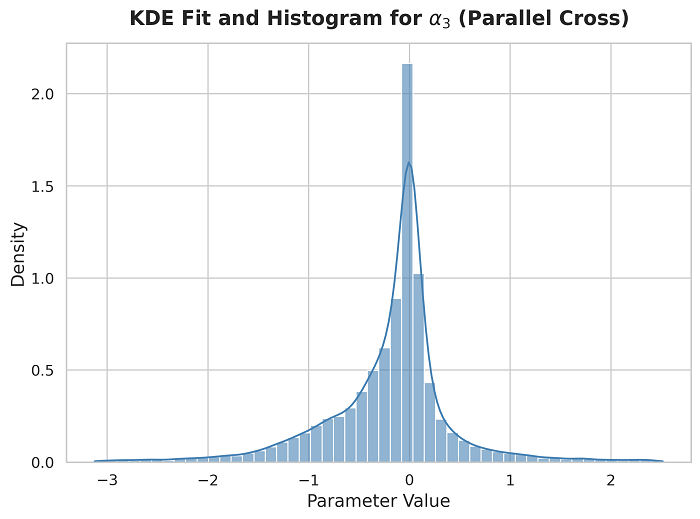

Supplement: S7 Fig — This figure shows the empirical distribution and kernel density estimate of α_cross across posts. The distribution indicates heterogeneity in the extent to which activity from other cascades influences retweet dynamics, suggesting that cross-cascade interactions vary substantially across content. (TIF) [file pone.0354472.s007.tif]

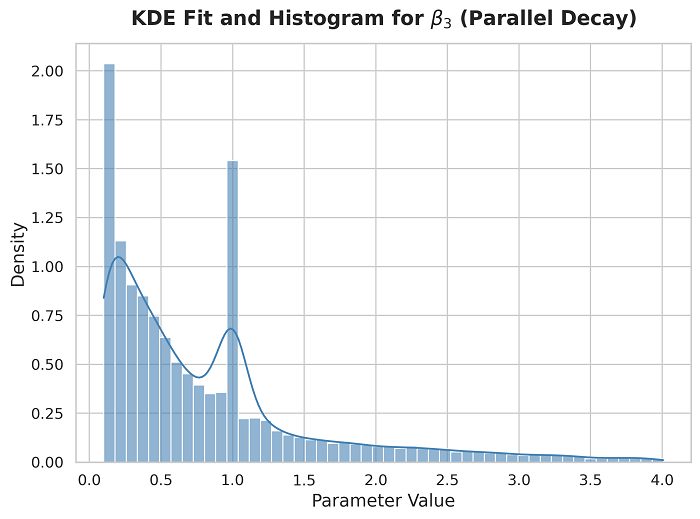

Supplement: S8 Fig — This figure shows the empirical distribution and kernel density estimate of β_cross across posts. The distribution indicates variation in how quickly the influence of parallel information cascades on retweet activity decays over time. (TIF) [file pone.0354472.s008.tif]
